# The iron chelator deferasirox induces apoptosis by targeting oncogenic Pyk2/β-catenin signaling in human multiple myeloma

**DOI:** 10.18632/oncotarget.11830

**Published:** 2016-09-02

**Authors:** Yusuke Kamihara, Kohichi Takada, Tsutomu Sato, Yutaka Kawano, Kazuyuki Murase, Yohei Arihara, Shohei Kikuchi, Naotaka Hayasaka, Makoto Usami, Satoshi Iyama, Koji Miyanishi, Yasushi Sato, Masayoshi Kobune, Junji Kato

**Affiliations:** ^1^ Department of Medical Oncology and Hematology, Sapporo Medical University School of Medicine, Japan

**Keywords:** deferasirox, Pyk2, β-catenin, apoptosis, multiple myeloma

## Abstract

Deregulated iron metabolism underlies the pathogenesis of many human cancers. Recently, low expression of ferroportin, which is the only identified non-heme iron exporter, has been associated with significantly reduced overall survival in multiple myeloma (MM); however, the altered iron metabolism in MM biology remains unclear. In this study we demonstrated, by live cell imaging, that MM cells have increased intracellular iron levels as compared with normal cells. In experiments to test the effect of iron chelation on the growth of MM cells, we found that deferasirox (DFX), an oral iron chelator used to treat iron overload in clinical practice, inhibits MM cell growth both *in vivo* and *in vitro*. Mechanistically, DFX was found to induce apoptosis of MM cells via the inhibition of proline-rich tyrosine kinase 2 (Pyk2), which is known to promote tumor growth in MM. Inhibition of Pyk2 is caused by the suppression of reactive oxygen species, and leads to downregulation of the Wnt/β-catenin signaling pathway. Taken together, our findings indicate that high levels of intracellular iron, which might be due to low *ferroportin* expression, play a role in MM pathophysiology. Therefore, DFX may provide a therapeutic option for MM that is driven by deregulated iron homeostasis and/or Pyk2/Wnt signaling.

## INTRODUCTION

Iron is essential for many fundamental cellular functions, including proliferation and DNA synthesis [1]. Accumulating evidence suggests that abnormal iron metabolism plays an important role in carcinogenesis and in the progression of many tumors [2]. In cancer cells, the demand for iron increases in response to sustained, accelerated cell proliferation and DNA synthesis [3].

Multiple myeloma (MM) is characterized by clonal proliferation of long-lived plasma cells within the bone marrow. Despite recent advances in its treatment, MM remains an incurable disease, underlining the continuing need to explore its molecular characteristics [4]. Recently, two independent groups reported that serum ferritin, which is used as a marker for iron overload, can be a negative prognostic indicator in MM [5, 6]. However, the mechanisms underlying this effect have not been clarified, and the significance of iron metabolism in MM cells remains unclear. Ferroportin has also been shown to have a role in MM progression [7]. This cell-surface transmembrane protein is the only non-heme iron exporter identified in mammalian cells, and is a pivotal protein of iron homeostasis [8]. Reduced expression of ferroportin can lead to intracellular iron overload.

We previously reported that excess iron is a feature of myelodysplastic syndrome (MDS) and hepatocellular carcinoma pathogenesis [9, 10]. Moreover, we found that iron chelation with deferasirox (DFX) suppresses leukemic transformation in MDS patients, and long-term phlebotomy and low iron diet therapy lower the risk of hepatocarcinogenesis in patients with chronic hepatitis C. Remarkably, iron chelation therapy has been shown to suppress cancer cell growth and is considered to be a promising cancer treatment [11]. Several mechanisms for the cytotoxic effects of iron chelation therapy have been reported; however, its action might depend on both the cell type and the iron chelation agent [12–14].

Proline-rich tyrosine kinase 2 (Pyk2) is a member of the focal adhesion kinase (FAK) family. It is a non-receptor protein kinase that shares a similar central domain with FAK, showing 60% sequence identity. In addition, Pyk2 has a conserved arrangement of proline-rich regions and tyrosine phosphorylation sites [15]. Pyk2 plays a crucial role in cell proliferation, migration and invasion in several cancers [16]. Specifically, it has been implicated in the progression of MM and in micro-environment-specific MM cell survival [17, 18]. Hence, it is an attractive target for MM therapeutics.

In the present study, we observed that reduced *ferroportin* mRNA levels might lead to increased intracellular iron concentrations in MM cells. These observations prompted us to investigate the effects of iron chelation with DFX on MM. Both *in vitro* and *in vivo*, studies demonstrated that DFX induces apoptosis via the inhibition of Pyk2/β-catenin signaling in MM. Overall, our findings reveal that dysregulation of iron metabolism is a characteristic of MM and provide a strong rationale for using DFX as a therapeutic Pyk2/β-catenin inhibitor to treat MM.

## RESULTS

### Expression of *ferroportin* correlates with clinical outcomes

**Figure 1:**
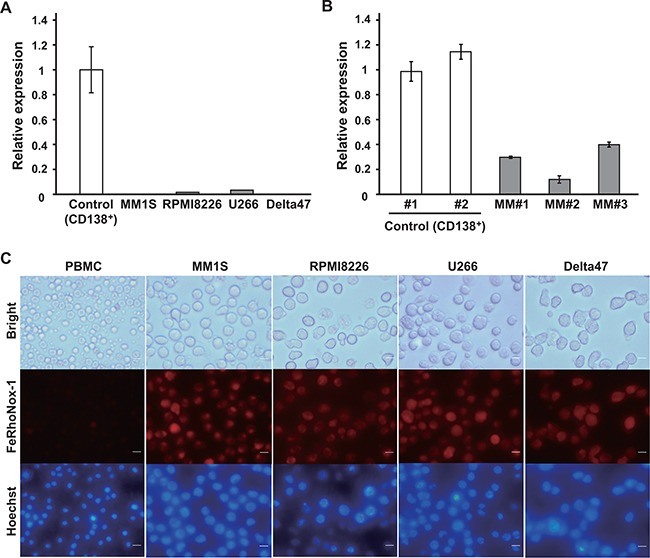
Decreased *ferroportin* expression and intracellular iron accumulation in MM cells **A, B.** Expression of *ferroportin* mRNA in MM cell lines (A) and in CD138^+^ cells from MM patients (B) evaluated by qRT-PCR. Data are the mean of triplicate measurements. Error bars represent the SD. **C.** Intracellular iron content was monitored by live cell microscopy. Intracellular Fe^2+^ was stained by a FeRhoNox-1 probe. Scale bars, 10 μM.

We previously demonstrated that an excess of intracellular iron contributes to the pathogenesis of MDS and hepatocellular carcinoma [9, 10]. To explore the hypothesis that iron overload might be also associated with MM pathogenesis, we initially analyzed the prognostic relevance of genes related to iron metabolism (Supplementary Tables S1 and S2) utilizing a data set in the public domain containing microarray profiles and MM patient outcomes. As Gu et al. [7] have shown, transcript levels of *ferroportin* are correlated with event-free survival (EFS) and overall survival (OS) in this data set, with a statistically significant negative correlation between *ferroportin* levels and EFS or OS (Supplementary Figure S1A, S1B). We therefore evaluated *ferroportin* mRNA expression in MM cell lines and primary MM cells. Quantitative reverse transcription (qRT)-PCR studies showed lower *ferroportin* mRNA expression in both MM cell lines and primary MM cells as compared with control cells (Figure 1A, 1B).

We also measured *ferroportin* transcript levels in other hematological malignancies, including leukemia and lymphoma. *ferroportin* mRNA expression was lower in B cell malignant cells than in leukemic cells or normal peripheral blood mononuclear cells (PBMCs) from healthy volunteers (Supplementary Figure S2).

### Increased intracellular iron in MM cells

We considered that low expression of *ferroportin* might result in an increase in intracellular iron. To examine intracellular iron levels in MM cell lines, we determined the cellular iron content using FeRhoNox-1 staining [19]. Live cell imaging showed that cellular iron levels were apparently higher in MM cell lines than in PBMCs from healthy volunteers (Figure 1C). Together with another study [7], our data suggested that excess intracellular iron might contribute to MM pathogenesis.

### Inhibition of MM cell proliferation by DFX

**Figure 2:**
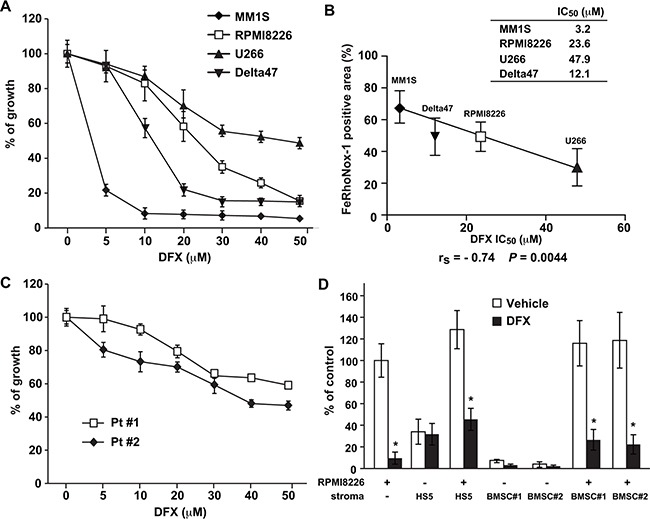
Iron chelation with DFX inhibits the proliferation of cultured MM cells **A, C.** MM cell lines (A) and primary MM cells isolated from two MM patients (C) were cultured with DFX (0-50 μM) for 48 hours. Cell proliferation was assessed in triplicate cultures by WST-1 assays. Data are the mean ± SD of triplicate measurements (n = 3; *P* < 0.05 for all cell lines). IC_50_ values are for growth inhibition by DFX. (B) Correlation between the FeRhoNox-1-positive area and IC_50_ values for DFX in four MM cell lines. The relationship was tested by two-tailed Spearman correlation (r_s_). **D.** RPMI8226 cells were cultured for 48 hours in HS-5-coated, primary BMSC-coated, or uncoated wells with DFX at its IC_50_ concentration. Primary BMSCs were derived from two MM patients (#1, #2). Cell proliferation was assessed by BrdU assays. Data are the mean of triplicate measurements. Error bars represent the SD. * *P* < 0.01.

Our observations suggested that iron chelation might be an effective therapeutic intervention against MM. To examine whether DFX, an oral, long-acting iron chelator that is approved for iron overload, might be effective in MM, we tested its influence on the proliferation of MM cell lines using WST-1 assays. DFX significantly reduced the proliferation of MM cell lines, with IC_50_ values ranging from 3.2 to 47.9 μM (Figure 2A, 2B).

To assess the threshold level of intracellular iron for predicting DFX-induced cytotoxicity, the correlation between intracellular iron content and the IC_50_ for DFX was investigated in four MM cell lines. As shown in Figure 2B, a significant correlation was observed between the FeRhoNox-1-positive area and the IC_50_ of DFX (r_s_ = −0.74, *P* = 0.004). Next, because bone marrow stromal cells (BMSCs) promote MM cell survival and induce drug resistance in MM cells, we tested the effect of BMSCs on the sensitivity of MM cells to DFX treatment. As shown in Figure 2D, DFX overcame the survival advantage conferred by the bone marrow microenvironment.

### DFX induces apoptosis in human MM cells

**Figure 3:**
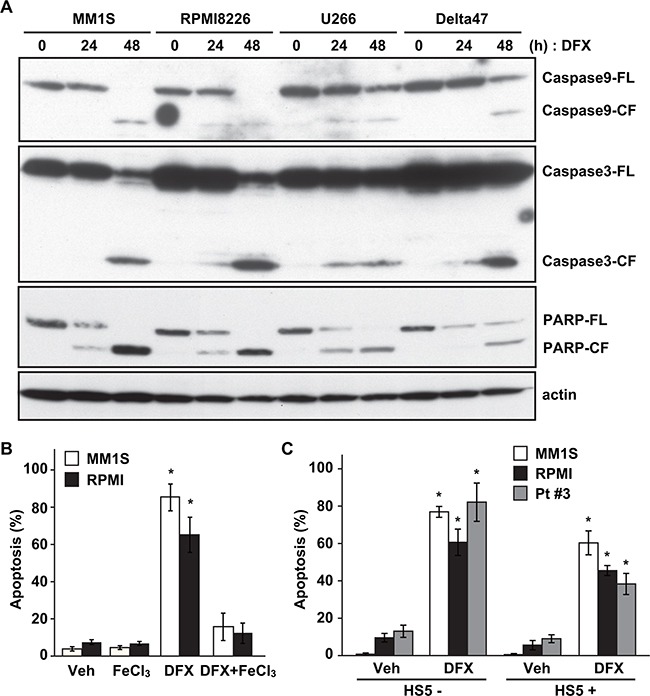
DFX induces apoptosis in MM cells **A.** Four MM cell lines were treated with DFX for 0, 24 and 48 hours. Whole-cell lysates were subjected to immunoblotting using anti-Caspase 9, anti-Caspase 3, anti-PARP, and anti-β-actin antibodies. FL; full length, CL; cleaved form. **B.** MM1S and RPMI8226 cells were pre-incubated with or without 100 mM FeCl_3_ for 2 hours. Cells were washed three times with phosphate-buffered saline and then treated with the IC_50_ concentration of DFX for 48 hours. Apoptotic cells were analyzed by flow cytometry using Annexin V/7-AAD staining. Data are the mean of triplicate measurements. Error bars represent the SD. * *P* < 0.01. **C.** MM1S, RPMI8226 and primary MM (#3) cells were cultured for 48 hours in BMSC (HS-5)-coated or uncoated wells with DFX. Apoptotic cells were analyzed by flow cytometry using Annexin V/7-AAD staining. Data are the mean of triplicate measurements. Error bars represent the SD. * *P* < 0.01.

To elucidate the cytotoxic mechanism of DFX on MM cells, we examined proteins involved programmed cell death by immunoblotting and performed flow cytometry analysis. We observed increased proteolytic cleavage of Caspase 9, Caspase 3, and PARP, but not Caspase 8 (data not shown) in all MM cell lines (Figure 3A), indicating cell apoptosis. Treatment with the pan-Caspase inhibitor Z-VAD significantly inhibited DFX-induced apoptosis (Supplementary Figure S3). These results indicated that the cytotoxicity triggered by DFX is mediated, at least in part, via caspase-dependent (intrinsic) apoptosis.

As shown in Figure 3B, Annexin V/7-AAD staining showed a markedly higher percentage of apoptosis among MM1S and RPMI8226 cells (Figure 3B). A previous study has shown that DFX induces autophagy in MM cells [20]. In the present study, however, autophagic cell death was not indicated by immunoblotting of LC3A/B (data not shown).

To assess the contribution of iron chelation by DFX to cytotoxicity, we investigated whether iron supplementation might affect apoptosis. Pretreatment of MM cells with ferric chloride (FeCl_3_, 100 μM) in combination with DFX prevented DFX-induced apoptosis, as evaluated by flow cytometry (Figure 3B). These observations strongly indicate that DFX-induced apoptosis depends on iron chelation by DFX. Furthermore, DFX-induced apoptosis was detected in MM cell lines and primary MM cells (#3) under co-culture systems with BMSCs (Figure 3C).

### DFX inhibits phosphorylation of Pyk2

**Figure 4:**
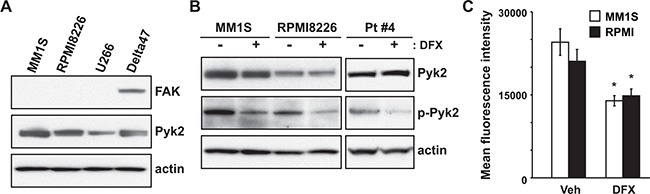
DFX exerts anti-myeloma activity by inhibition of Pyk2 phosphorylation accompanied by reduced production of ROS **A.** Expression of FAK and Pyk2 in MM cell lines was evaluated by immunoblotting. **B.** Immunoblot of total protein extracts obtained from MM1S, RPMI8226 and primary MM cells (#4) treated with or without DFX for 24 hours. The active form of Pyk2 was analyzed by using an anti-p-Pyk2 antibody. **C.** MM1S and RPMI8226 cells were cultured for 2 hours with DFX. ROS production was analyzed by flow cytometry using CellRox deep Red staining. Data are the mean of triplicate measurements. Error bars represent the SD. * *P* < 0.01.

To clarify the mechanisms of DFX-induced apoptosis in MM, we analyzed components of the phosphoinositide 3-kinase (PI3K)/Akt signaling pathway by PCR array (Qiagen, Hilden, Germany), because this pathway plays a crucial role in MM pathogenesis [21]. Unexpectedly, as shown in Supplementary Figure S4, *FAK* transcript expression was substantially suppressed after DFX treatment in both MM1S and RPMI8226 cells. Expression of FAK protein was therefore tested by immunoblotting in a panel of MM cells. However, no expression of FAK protein was observed in three of the four cell lines (Figure 4A), indicating that FAK is not associated with DFX-induced apoptosis.

Next, we investigated Pyk2, which is a member of the FAK family and shares 48% amino acid sequence identify with FAK. Pyk2 was expressed differentially in the four different MM cell lines (Figure 4B). Consistent with a previous report [17], basal Pyk2 expression was relatively low in U266 cells. Consequently, we assessed Pyk2 activation after treatment with DFX. DFX significantly reduced the level of phosphorylated Pyk2(Tyr402), indicating in a loss of Pyk2 activity (Figure 4B). There were no substantial differences in total Pyk2 levels. In addition, we assessed the janus kinases Jak1, Jak2 and Jak3, because these molecules are also relevant in MM pathogenesis [22]. As shown Supplementary Figure S5, *Jak1*, *Jak2* and *Jak3* transcript levels were not suppressed by DFX treatment.

Recently, it was reported that reactive oxygen species (ROS) induce phosphorylation of Pyk2 in acute monocytic leukemia cells [23]. Furthermore, we have previously shown that DFX can inhibit ROS production in MDS [9]. These two observations prompted us to analyze changes in ROS production in DFX-treated MM cells. Flow cytometric analysis using CellROX deep red probes revealed that DFX significantly suppressed ROS production in MM cells (Figure 4C). Taken together, these data suggest that DFX exerts anti-myeloma activities by inhibiting Pyk2 phosphorylation, following a decrease in ROS production.

### Pyk2 inhibition by DFX suppresses the Wnt/β-catenin signaling pathway

**Figure 5:**
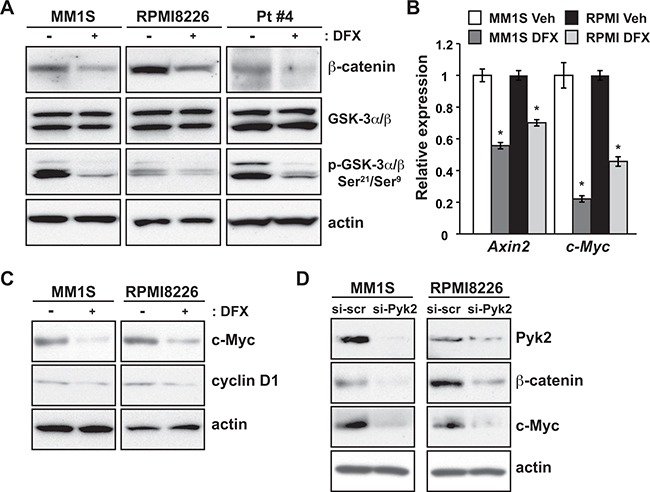
DFX treatment and Pyk2 silencing lead to suppression of the Wnt/β-catenin signaling pathway **A.** MM1S, RPMI8226 and primary MM cells were treated with DFX for 24 hours, and subjected to immunoblot analysis for total GSK3β and β-catenin protein. The inactive form of GSK3β was analyzed by using an anti-p-GSK3β antibody. **B.** qRT-PCR validation of pivotal Wnt target genes in MM1S and RPMI8226 cells treated with DFX. Data are the mean of triplicate measurements. Error bars represent the SD. * *P* < 0.01. **C.** Immunoblot analysis of c-Myc and cyclin D1 levels in DFX-treated MM cells. **D.** Immunoblot analysis of β-catenin and c-Myc protein expression in cells transfected with Pyk2 siRNA.

Next, we analyzed Pyk2 downstream target molecules in order to clarify the mechanism of DFX-induced apoptosis in MM. It has been shown that inhibition of Pyk2 promotes β-catenin degradation via the activation of GSK-3β [17]. In MM cells, as expected, β-catenin protein levels were reduced by DFX treatment, accompanied by decreased levels of phosphorylated-GSK-3β (inactive form), resulting in the degradation of β-catenin (Figure 5A).

Wnt signaling activities were also examined by qRT-PCR and immunoblotting. Treatment with DFX reduced the mRNA levels of *Axin2* and *c-Myc*, which are target genes of Wnt; in particular, *Axin2* is a robust and specific Wnt target gene (Figure 5B). In addition, immunoblotting showed that c-Myc and cyclin D1, which function downstream of β-catenin in the Wnt pathway, were significantly decreased by DFX treatment (Figure 5C). Collectively, these results supported the hypothesis that DFX inhibits activation of Wnt/β-catenin signaling.

Lastly, to pinpoint the function of Pyk2 in MM cells, we performed *in vitro* Pyk2 loss-of-function analysis. Similar to DFX treatment, Pyk2 knockdown with siRNA in MM cells led to a decrease in β-catenin and c-Myc protein levels (Figure 5D).

### Anti-tumor activity of DFX in mouse xenograft models

**Figure 6:**
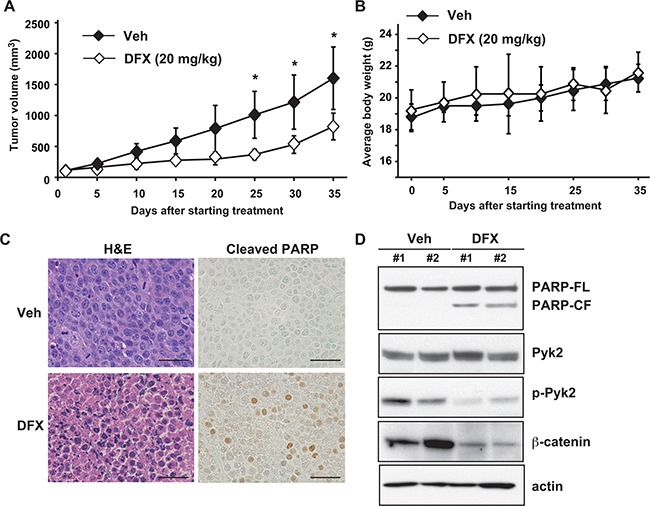
DFX inhibits tumor growth in a mouse xenograft model of MM **A.** Tumor volumes were calculated from caliper measurements. Data represent the mean ± SD (n=5). * *P* < 0.05. **B.** The body weight of mice treated with DFX or vehicle was monitored every day. Data are the mean ± SD. NS, not significant. **C.** Histologic analysis of sections stained with hematoxylin and eosin (H&E) and Cleaved-PARP antibody from DFX-treated mice. Scale bars, 50 μM. **D.** Tumors harvested from DFX-treated and untreated mice were subjected to immunoblot analysis for PARP, Pyk2, p-Pyk2 and β-catenin protein.

To test the therapeutic potential of DFX, we evaluated its ability to suppress tumor growth *in vivo* by using the subcutaneous RPMI8226 murine xenograft model of human MM. After DFX treatment, the MM tumor burden was significantly lower in the DFX group than in the vehicle group (Figure 6A). DFX treatment for 35 days was well tolerated, without causing significant body weight loss (Figure 6B). We also detected an increase in apoptotic tumor cells in mice treated with DFX as compared with vehicle-treated mice, as evaluated by Cleaved-PARP staining and immunoblotting (Figure 6C, 6D). No histologic changes in normal tissues were observed in the treatment group upon necropsy (Supplementary Figure S6).

To evaluate the inhibition of target proteins by DFX *in vivo*, we performed immunoblotting analysis of the tumors. Consistent with the *in vitro* results, significantly lower levels of phosphorylated Pyk2 and β-catenin protein were observed in tumors obtained from DFX-treated mice than in those obtained from vehicle-treated mice (Figure 6D). Taken together, these data demonstrate the potent *in vivo* anti-MM activity of DFX, providing the framework for clinical application of DFX in this disease.

To test whether the anti-proliferative effect of DFX might synergize with other agents commonly used to treat MM, we also conducted a study of combination treatment. Notably, the cytotoxic effects of bortezomib on MM cells were enhanced by DFX treatment (Supplementary Figure S7), suggesting the potential clinical utility of this combination therapy.

## DISCUSSION

In the current study, our data demonstrate that ferroportin plays an important role in the clinical outcome of MM. In the gene expression profiling of MM patients, we observed a reduction in *ferroportin* mRNA, which was associated with a poor prognosis. Low transcript levels of *ferroportin* were observed both in MM cell lines and in primary MM cells, which led us to evaluate intracellular iron in MM cells, because reduced expression of *ferroportin* might increase intracellular iron content [1, 24]. Previous studies also support this possibility, because MM cells have been shown to be able to uptake and store iron as well as normal plasma cells [25, 26]. Gu et al. [7] reported recently that down-regulated ferroportin correlated negatively with OS, as shown in the present study, and that the increase in intracellular iron content caused by reduced expression of ferroportin accelerated MM cell growth and bone absorption. However, iron homeostasis and intracellular iron accumulation in human MM cells has not yet been studied extensively. We are the first to demonstrate high concentrations of intracellular iron (Fe^2+^) by live cell imaging using the FeRhoNox-1 stain, providing insight into the role of intracellular iron overload in MM pathogenesis.

We next evaluated the effect of iron chelation as a treatment for MM, because excess iron can accelerate tumor cell proliferation. To date, DFX has been reported to have anti-tumor activity in several types of cancer [14, 27–29]. Indeed, *in vitro* results reported by Pullarkat *et al.* [20] during preparation of this manuscript indicated that DFX had a cytotoxic effect against MM cells. Consistent with this, our data revealed that DFX effectively induces apoptosis of MM tumor cells both *in vivo* and *in vitro*. Of note, intracellular iron content might predict whether MM cells will show DFX cytotoxicity.

Our data suggested that DFX suppressed ROS production and led to Pyk2 inhibition. Consequently, DFX-triggered apoptosis was initiated. In support of our findings, recent studies have shown that Pyk2 inhibitors might offer a novel therapeutic intervention for MM [17, 18]. Pyk2 inhibition results in suppression of Wnt signaling through the acceleration of β-catenin degradation caused by GSK3β activation [17]. Aberrant activation of the Wnt pathway plays a significant role in MM progression and maintenance [30–32]. Interestingly, iron chelators have been shown to act as Wnt inhibitors and to suppress tumor cell growth [33, 34]; however, the mechanism underlying the decreased expression of β-catenin protein caused by DFX has not been clearly defined. Notably, our findings provide the first description of the iron chelator DFX inhibiting MM cell growth via the inhibition of ROS/Pyk2/β-catenin.

We also demonstrated for the first time the efficacy of DFX in suppressing tumor growth *in vivo* by using a well-established murine xenograft model of MM [35]. Mice were treated daily with DFX at 20 mg/kg, a dosage that is clinically appropriate because it is the starting dose for patients with transfusional iron overload [36]. Therefore, our observations might be quickly translated into a clinical trial for MM patients, because DFX is well tolerated and is already approved and used widely to treat iron overload patients. Notably, we showed that administration of DFX also enhanced bortezomib cytotoxicity against MM cells. Bortezomib has been shown to markedly improve the EFS and OS for MM patients. However, both resistance to and adverse effects of bortezomib are emerging as therapeutic problems. Combination approaches have been proposed to prevent and/or overcome bortezomib resistance [37]. Accordingly, a combination therapy using DFX and bortezomib might provide a new strategy to enhance bortezomib cytotoxicity and overcome resistance to this MM therapeutic.

In practical terms, nephrotoxicity is acknowledged as the most common adverse effect of DFX. On the one hand, DFX nephrotoxicity, defined as an increase in serum creatinine level, has been observed in 18%–22% of DFX-treated patients [38]. Although the molecular mechanism underlying the nephrotoxicity remains unclear, it is characterized by proximal tubular dysfunction and related to reversible tubular cell injury. When this toxicity occurs, adjustment of the DFX dose should be considered. On the other hand, renal impairment known as “myeloma kidney” is a common complication of patients with MM, and 79% of patients have been found to have serum creatinine levels lower than 2 mg/dL at the time of diagnosis [39]. Bortezomib-based therapies were reported to bring a significant improvement of renal function (major renal response) to 77% of newly diagnosed MM patients [40]. Although the clinical administration of DFX to MM patients might pose some difficulties, DFX can be administered to MM patients with or without dose-adjustment protocols during the course of the disease.

In conclusion, this study has demonstrated that low expression of *ferroportin* is associated with a poor prognosis in MM and might be related to increased levels of intracellular iron that contribute to MM pathogenesis. Our results suggest the potential clinical application of DFX to the treatment of iron-overloaded and/or Pyk2/β-catenin-driven MM.

## MATERIALS AND METHODS

### Gene expression and survival analysis

Expression levels of iron regulatory genes (Supplementary Tables S1 and S2) were evaluated by using the publicly accessible MAQC-II Project MM data set (GSE24080) from the Gene Expression Omnibus. The correlation between ferroportin mRNA levels and clinical outcome was investigated by evaluating expression levels stratified by the median value. In total, 554 patients were categorized as having high or low levels of ferroportin, and Kaplan-Meier survival analysis comparing the two groups was performed.

### Reagents and human cell lines

DFX was obtained from Santa Cruz Biotechnology (Dallas, TX). Stock solutions were generated by dissolving the lyophilized powder in 100% DMSO at 100 mM. MM1S, RPMI8226 and U266 cell lines were kindly provided by Dr Ruben D. Carrasco (Dana-Farber Cancer Institute, Harvard Medical School, Boston, MA). Delta-47 and HS-5 cells were purchased from ATCC (Manassas, VA). All MM cell lines were cultured in RPMI 1640 (Sigma-Aldrich, St. Louis, MO) containing 10% fetal bovine serum (FBS), 2 μM l-glutamine and 1% penicillin-streptomycin. HS-5 cells were cultured in DMEM (Sigma-Aldrich) supplemented with 10% FBS, 2 μM l-glutamine and 1% penicillin-streptomycin.

### Patient specimens

PBMCs were obtained from blood samples from healthy volunteers by using Histopaque-1077 (Sigma-Aldrich) gradient fractionation. Bone marrow specimens were obtained from patients with MM after obtaining Sapporo Medical University School of Medicine Review Board approval and informed consent, and were obtained in compliance with the Declaration of Helsinki. Primary CD138^+^ plasma cells were purified by positive selection with anti-CD138 magnetic-activated cell separation microbeads (Miltenyi Biotec, Auburn, CA) as described [41]. According to a previous report, primary BMSCs were established from CD138^−^ bone marrow mononuclear cells [35].

### RNA extraction and quantitative reverse transcription-PCR

RNA was extracted with TRIzol Reagent (Life Technologies, Carlsbad, CA) in accordance with the manufacturer's protocol. Total RNA (1 μg) was reverse-transcribed by using a SuperScript VILO cDNA synthesis kit (Life Technologies), and qRT-PCR was performed with an Applied Biosystems 7300 Real-time PCR system (Applied Biosystems, Foster City, CA). Analysis of target genes was conducted in quadruplicate using the POWER SYBR Green Master Mix (Life Technologies) as previously described [30]. Transcripts levels were normalized to *β-actin* expression. The experiments were repeated three times. The following PCR primers were designed: 5′-CTACTTGGGGAGATCGGATGT-3′ and 5′-CTGGGC CACTTTAAGTCTAGC-3′ for *ferroportin*; 5′-AGCCA AAGCGATCTACAAAAGG-3′ and 5′-GGTAGGCAT TTTCCTCCATCAC-3′ for *Axin2*; 5′-TTTTTCGGGT AGTGGAAAACC-3′ and 5′-GCAGTAGAAATACGGC TGCAC-3′ for *c-Myc*; 5′-GGCATCCTCACCCTGA AGTA-3′ and 5′-GAAGGTGTGGTGCCAGATTT-3′ for *β-actin*.

### Fe^2+^ fluorescent stain

For Fe^2+^ detection, cells were stained for 1 hour at 37°C with 5 μM FeRhoNox-1 (Goryo Chemical, Sapporo, Japan) and 1 μg/ml hoechst33342 (Life Technologies). After staining, cells were washed and observed by live cell imaging using a Biozero BZ-8000 fluorescence microscope (KEYENCE Laboratories, Osaka, Japan).

### Growth inhibition and apoptosis assays

The inhibitory effect of DFX on MM cell line growth was assessed by WST-1 assay, as described previously [42]. To analyze the proliferation of MM cells with or without BMSCs, the rate of DNA synthesis was determined by BrdU assay (BrdU cell proliferation assay reagent, Cell Signaling Technology, Danvers, MA). Apoptosis was evaluated by PARP immunoblotting and quantified by using an Annexin V/7-AAD staining kit in accordance with the manufacturer's instructions (Annexin V PE Apoptosis Detection Kit I, BD Biosciences, San Jose, CA), followed by analysis on a BD FACS Canto II instrument using FACSDiva (BD Biosciences) [43].

### Inhibition of Pyk2 expression by small-interfering RNA (siRNA)

A non-silencing control and siRNA targeting Pyk2 were purchased from Thermo Scientific (Waltham, MA) and transfected into cell lines by electroporation using Nucleofector, according to a previous report [18].

### Immunoblotting

MM cells were cultured with or without stimuli; the cells were then harvested, washed, lysed, and stained by using primary antibodies to the following proteins: PARP (#9542), FAK (#3285), Pyk2 (#3292), Phospho-Pyk2 (Tyr402) (#3291), GSK-3-α/β (#5676), p-GSK-3-α/β (Ser^21^/Ser^9^) (#9331), cyclin D1 (#2978) (all Cell Signaling Technology); c-myc (OP10L; EMD Biosciences, San Diego, CA); Actin-HRP (sc-1615; (Santa Cruz Biotechnology); β-catenin (CAT5-H10; Invitrogen Corporation, Carlsbad, CA). Standard chemiluminescence was used to evaluate protein expression.

### ROS measurement

For quantification of ROS, cells were stained for 30 min at 37°C with 5 μM CellROX Deep Red (Invitrogen). After staining, cells were washed and suspended in PBS. For quantification of intracellular ROS, cells were analyzed by a BD FACS Canto II instrument (BD Biosciences, Tokyo, Japan).

### Murine xenograft model of human MM

NOD/Shi-scid IL-2γnul (NOG) mice (8 weeks old, female) were purchased from the Central Institute for Experimental Animals (Kawasaki, Japan). All animal studies were conducted according to protocols approved by the Animal Ethics Committee of the Sapporo Medical University School of Medicine. Mice were inoculated subcutaneously in the left flank with 3 × 10^7^ RPMI8226 cells in 100 μL of RPMI1640 with 100 μL of Matrigel (Corning, Corning, NY). When the tumor volume reached 100 mm^3^, mice were assigned to two groups that received either vehicle alone or DFX (n = 5 per group). The DFX group received DFX suspended in vehicle [30% 1,2-propanediol/70% sterile 0.9% sodium chloride solution (v/v)], which was administered by oral gavage every day at 20 mg/kg [29]. Control mice were treated in the same way with vehicle only. Caliper measurements of the longest perpendicular tumor diameters were performed on alternate days to estimate the tumor volume via the following formula: length × width^2^ × 0.5. Complete necropsies were performed for each experimental animal. Tissues were subjected to hematoxylin and eosin staining, and immunohistochemical analysis with anti-Cleaved PARP antibody (Cell Signaling Technology).

### Statistical analysis

The significance of differences between groups was determined by Student *t* test. Statistical significance was defined at a *P* value of less than 0.05. Kaplan-Meier survival analysis was carried out by using GraphPad Prism analysis software version 6.0a (GraphPad Software, San Diego, CA).

## SUPPLEMENTARY FIGURES AND TABLES


