# Supplementary material for: The iron chelator deferasirox induces apoptosis by targeting oncogenic Pyk2/β-catenin signaling in human multiple myeloma
Source: Oncotarget. 2016 Sep 2;7(39):64330–41. doi: 10.18632/oncotarget.11830 (PMC5325446; doi:10.18632/oncotarget.11830)
Supplement: Supplementary file 1 [file oncotarget-07-64330-s001.pdf]

The iron chelator deferasirox induces apoptosis by targeting oncogenic Pyk2/β-catenin signaling in human multiple myeloma

SUPPLEMENTARY FIGURES AND TABLES

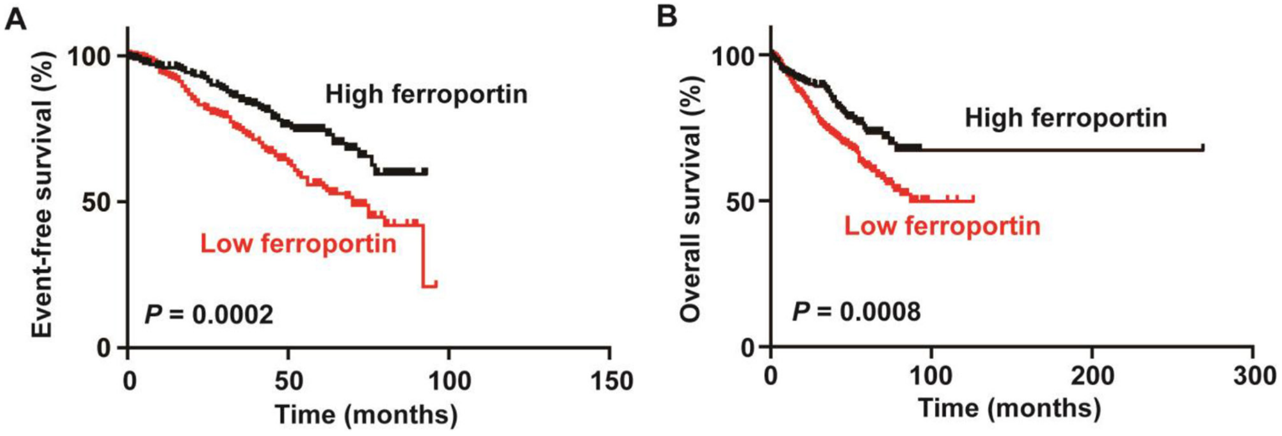

Supplementary Figure S1: *Ferroportin* expression in MM determines clinical outcome. Kaplan-Meier survival curves based on *ferroportin* expression in gene expression data sets. Patients were categorized as high or low expression of *ferroprtin* according to *ferroprtin* expression levels based on median partitioning. MM patients that are high expression of *ferroportin* have significantly better Event-Free survival **A.** and Overall survival **B.** than low-*ferrportin* expressed patients.

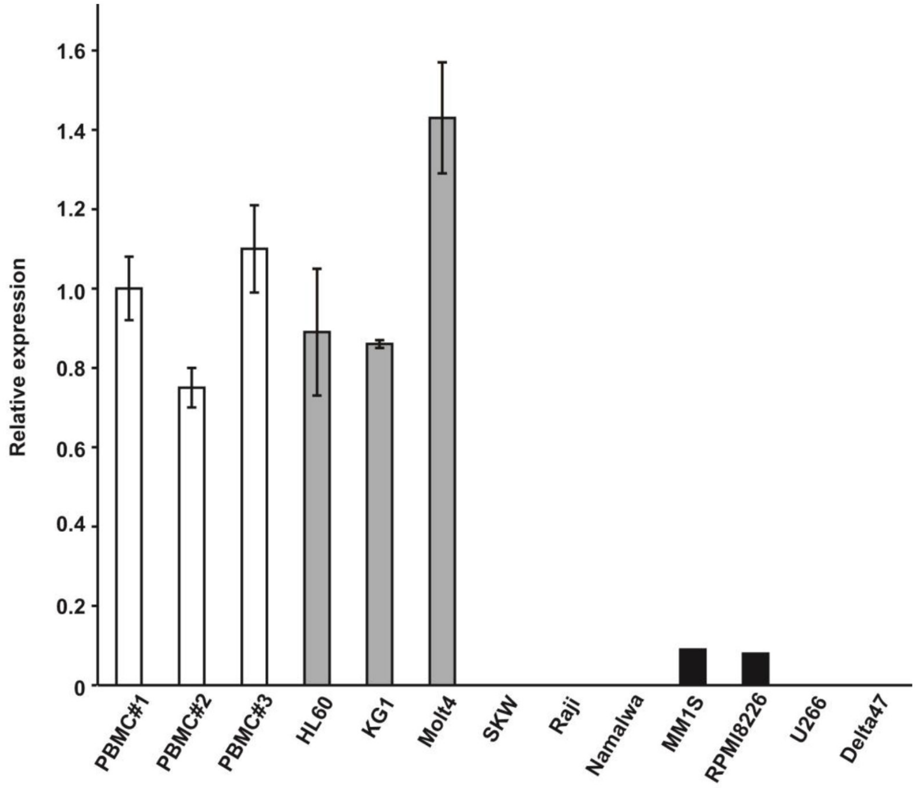

Supplementary Figure S2: *Ferroportin* expression in hematological malignancy cells. *Ferroportin mRNA* expression evaluated by qRT-PCR in several hematological malignancy cells. Data are mean ± SD of triplicate measurements. Error bars represent SD.

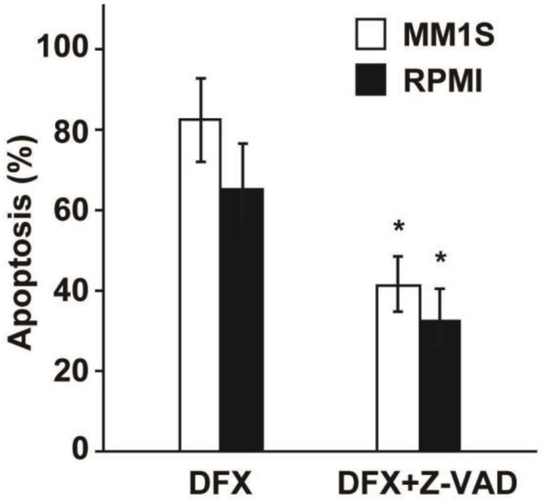

Supplementary Figure S3: A caspase inhibitor rescues DFX induced apoptosis in MM cells. MM1S and RPMI8226 cells were pre-incubated with or without 50μM z-VAD-fmk for 2 hours. Thereafter, cells were treated with IC_{50} level of DFX for 48 hours. Apoptotic cells were analyzed by flow cytometry using Annexin V/7-AAD staining. Data are mean ± SD of triplicate measurements. Error bars represent SD.

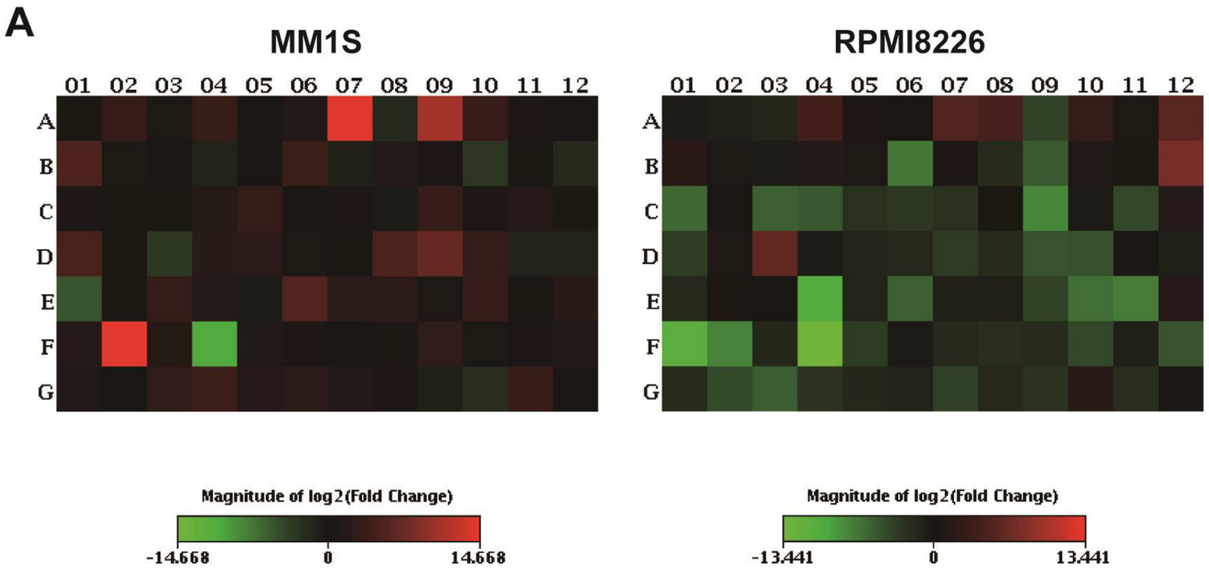

B

Position	RefSeq	Symbol	Description	Fold change (⊿⊿CT) MM1S	Fold change (⊿⊿CT) RPMI8226
F04	NM_005607	PTK2	PTK2 protein tyrosine kinase 2	5.01×10^{-4}	8.92×10^{-5}

Supplementary Figure S4: Expression levels of *mRNA* associated with PI3K-Akt signaling pathway in MM1S and RPMI8226 cells treated with DFX. A. Heat map representation of the 89 genes listed in columns (A-G) and in rows (1-12) - for the MM1S (left) and RPMI8226 (right) signatures validated with PCR array. **B.** A PTK2 gene information, which was the most down-regulated by DFX treatment.

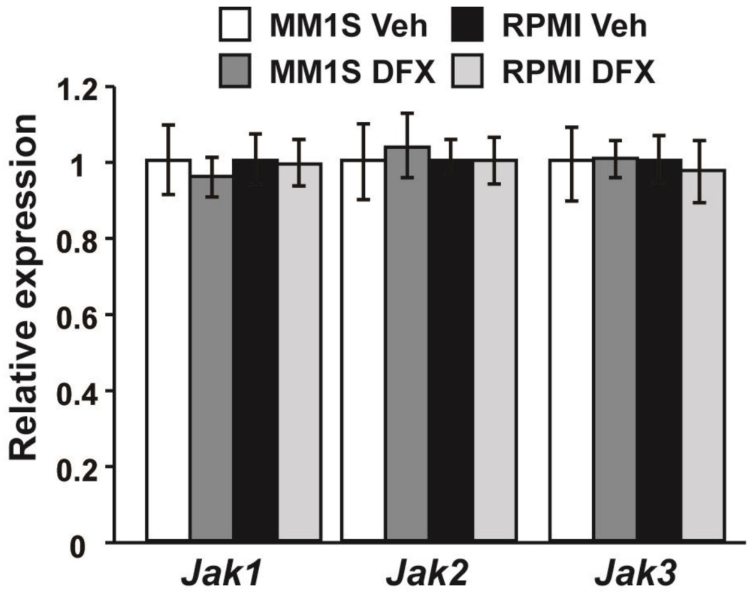

Supplementary Figure S5: *Jak1*, *Jak2* and *Jak3* expression in MM cells. qRT-PCR validation of *Jak1*, *2*, and *3* genes in MM1S and RPMI8226 cells treated with DFX. Data are mean ± SD of triplicate measurements. Error bars represent SD.

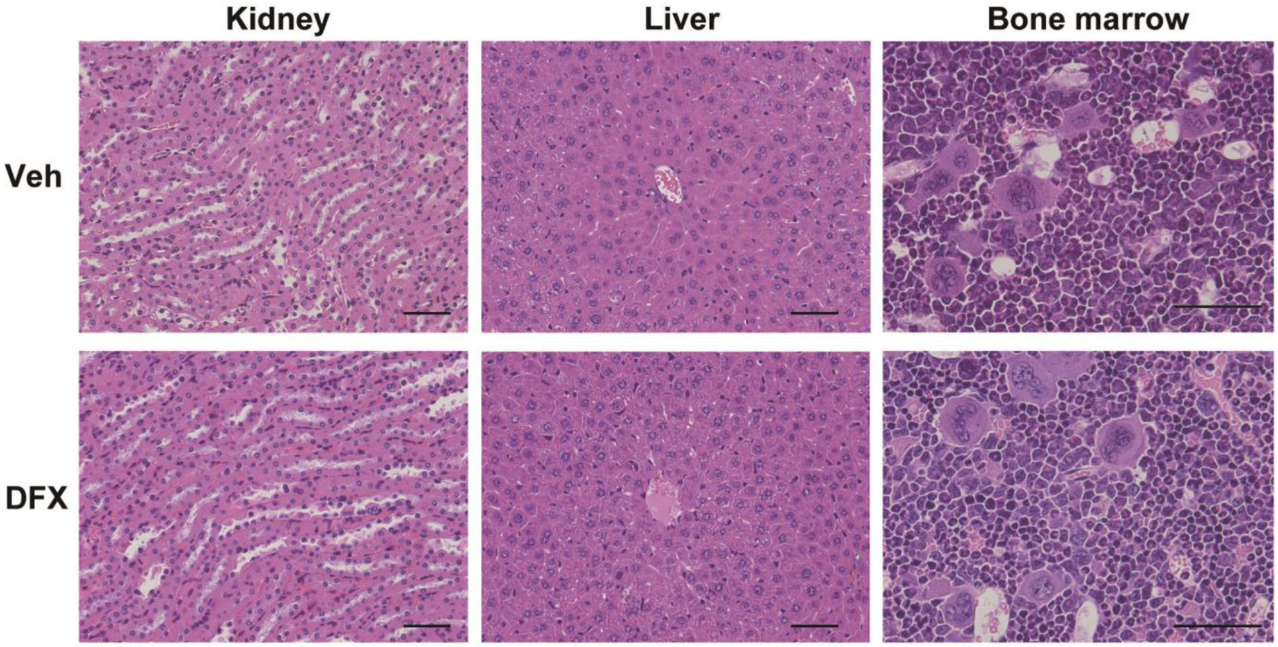

Supplementary Figure S6: Histology of liver, kidney and bone marrow of DFX-treated mice. H&E staining of liver, kidney and bone marrow tissues isolated from experimental mice injected with RPMI8226 cells and treated with vehicle or DFX showed no evidence of toxicity across all histologic specimens. Scale bar, 50µM. H&E, hematoxylin and eosin.

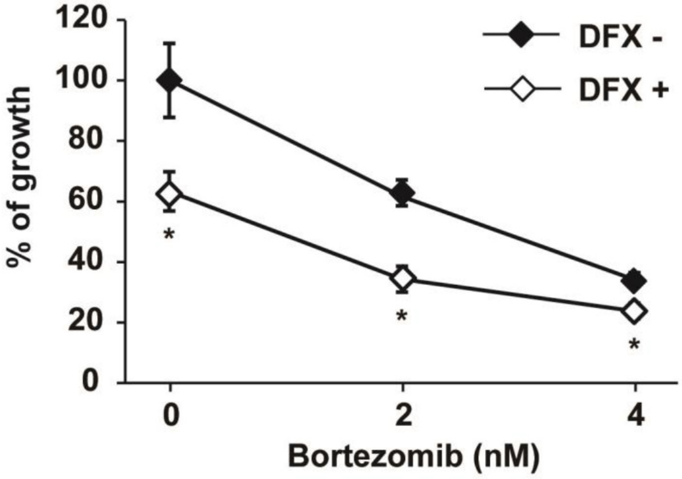

Supplementary Figure S7: DFX enhances cytotoxic effect induced by bortezomib. MM1S cells were cultured for 48 hours with increasing concentration of bortezomib in combination with 2 μM DFX. Cell growth was assessed by WST-1 assay of triplicate cultures, expressed as the percentage of untreated control. Data, mean ± SD. *$P < 0.01$.

Supplementary Table S1: The analyzed 11 iron regulatory genes for EFS

Probe set IDs	Symbol	Name	*P* *	HR **	95% CI
223044_at	SLC40A1	Solute carrier family 40 (iron-regulated transporter), member 1 (Ferroportin)	0.0002	0.60	0.41-0.76
222453_at	CYBRD1	Cytochrome b reductase 1	0.0177	0.69	0.51-0.94
220491_at	HAMP	Hepcidine antimicrobial peptide	NS	1.19	0.88-1.63
210047_at	SLC11A2	Sloute carrier family 11 (proton-coupled divalent metal ion transporters), member 2 (DMT-1)	NS	0.85	0.62-1.15
203902_at	HEPH	Hephaestin	NS	1.31	0.96-1.78
208691_at	TFRC	Transferrin receptor (p90, CD71)	NS	1.02	0.75-1.39
207883_s_at	TFR2	Transferrin receptor 2	NS	0.84	0.62-1.14
212788_x_at	FTL	Ferritin, light polypeptide	NS	0.77	0.56-1.04
214211_at	FTH1	Ferritin, heavy polypeptide 1	NS	0.78	0.58-1.07
207071_s_at	ACO1	Aconitase 1, soluble (IRP1)	NS	0.78	0.57-1.06
225892_at	IREB2	Iron-responsive element binding protein 2	NS	0.93	0.68-1.26

HR, hazard ratio; CI, confidence interval; NS, not significant
*Log-Rank test *P*.
**Values <1.0 indicate that expression in positively correlated with good survival.

Supplementary Table S2: The analyzed 11 iron regulatory genes for OS

Probe set IDs	Symbol	Name	*P* *	HR **	95% CI
223044_at	SLC40A1	Solute carrier family 40 (iron-regulated transporter), member 1 (Ferroportin)	0.0008	0.60	0.44-0.81
222453_at	CYBRD1	Cytochrome b reductase 1	0.0058	0.66	0.49-0.88
220491_at	HAMP	Hepcidine antimicrobial peptide	NS	0.98	0.72-1.33
210047_at	SLC11A2	Sloute carrier family 11 (proton-coupled divalent metal ion transporters), member 2 (DMT-1)	NS	1.12	0.83-1.51
203902_at	HEPH	Hephaestin	NS	1.00	0.74-1.35
208691_at	TFRC	Transferrin receptor (p90, CD71)	NS	1.24	0.92-1.68
207883_s_at	TFR2	Transferrin receptor 2	NS	0.96	0.71-1.30
212788_x_at	FTL	Ferritin, light polypeptide	NS	0.87	0.64-1.17
214211_at	FTH1	Ferritin, heavy polypeptide 1	NS	0.75	0.55-1.01
207071_s_at	ACO1	Aconitase 1, soluble (IRP1)	NS	0.82	0.60-1.10
225892_at	IREB2	Iron-responsive element binding protein 2	NS	1.12	0.89-1.62

HR, hazard ratio; CI, confidence interval; NS, not significant
*Log-Rank test *P*.
**Values <1.0 indicate that expression in positively correlated with good survival.
